# The Effects of Endothelial Protein C Receptor Gene Polymorphisms on the Plasma sEPCR Level in Venous Thrombosis Patients

**DOI:** 10.5152/tjh.2011.40

**Published:** 2012-03-05

**Authors:** Afife Karabıyık, Erkan Yılmaz, Yonca Eğin, Nejat Akar

**Affiliations:** 1 Ankara University, Biotechnology Institute, Ankara, Turkey; 2 Ankara University, Department of Pediatric Molecular Genetics, Ankara, Turkey

**Keywords:** Thrombosis, Endothelial protein C receptor (EPCR), Soluble EPCR (sEPCR), EPCR haplotypes

## Abstract

**Objective:** The aim of this study was to investigate variations in the endothelial cell protein C receptor gene (EPCRgene) that may play a role in thrombosis and the effects of these variations on the plasma soluble endothelial cell proteinC receptor (sEPCR) level in Turkish patients with venous thrombosis.

**Material and Methods:** This study included 111 thrombosis patients and 73 healthy controls. Following DNAextraction, PCR, SSCP, and DNA sequencing analysis of 4 exons of the EPCR gene was performed. Plasma sEPCR wasmeasured via enzyme-linked immunosorbent assay (ELISA).

**Results:** In all, 3 polymorphisms were detected in exons 1-4. C3998T (SNP no: rs2069952) polymorphism was detectedin intron 2 and C4678G (A1 haplotype) (SNP no: rs9574) in the 3’ untranslated region (3’UTR). There weren’t anysignificant differences in C3998T polymorphism between the control and patient groups. There wasn’t a significantdifference in plasma sEPCR levels between both controls and patients that carried the A1 allele. A4600G substitution (A3haplotype) (SNP no: rs867186) was observed in exon 4 and was associated with a 2.04-fold higher risk of thrombosis.A3 allele carriers had higher sEPCR levels than those without the allele. Mean sEPCR level in the patients with thehomozygous A3 allele was 289 ng μL–1, versus 113.42 ng μL–1 in those with the homozygous A1 allele.

**Conclusion:** The A1 haplotype might offer protection against thrombosis and the A3 haplotype might be associatedboth with elevated plasma sEPCR and elevated risk of venous thrombosis in the Turkish population. Plasma sEPCRlevels were significantly higher in those that carried the A3 allele (4600A>G) (both patients and controls). Among theparticipants that carried the A1 allele (4678C>G), plasma sEPCR did not differ significantly.

## Abstract (tr)

**Amaç:** Bu çalışmada, trombozda önemli bir role sahip olan EPCR genindeki varyasyonların tespit edilmesi ve bu varyasyonların Türk populasyonunda plazma sEPCR seviyeleri üzerindeki olası etkisinin incelemesi amaçlanmıştır.

**Gereç ve Yöntemler:** Çalışmamız 111 trombozlu olgu ve 73 sağlıklı kontrol içermektedir. EPCR geninin dört ekzonu intronlardan başlanan primerler kullanılarak PCR, SSCP ve DNA Dizi Analizi ile taranmıştır. Plazma sEPCR düzeyleri ELISA yöntemi kullanılarak ölçülmüştür.

**Bulgular: **Bu çalışmada, EPCR haplotipleri ile sEPCR seviyesi arasındaki ilişki incelenmiş ve EPCR geninin taranan dört ekzonunda üç farklı polimorfizm saptanmıştır. Bunlardan C3998T (rs2069952) genin 2. intronunda, A1 haplotipini oluşturan C4678G (rs9574) ise EPCR geninin 3’ UTR bölgesinde tespit edilmiştir. EPCR geninde tespit edilen diğer bir polimorfizm ise A3 haplotipini oluşturan ve ekzon 4’de yer alan A4600G (rs867186) değişimidir. A3 allelini homozigot olarak taşıyan hastalarda ortalama sEPCR seviyesi 289 ng/μl iken, A1 allelini homozigot olarak taşıyanlarda 113,42 ng/μl’dır.

**Sonuç:** İstatistik analizler kontrol ve hastalar arasında C3998T değişiminin tromboz riski açısından anlamlı olmadığını göstermiştir. A1 haplotipinin ise trombozdan koruyucu bir etkiye sahip olabileceği saptanmış ancak A1 (4678C>G) allelini taşıyan bireylerde sEPCR seviyesinde de anlamlı bir değişiklik saptanamamıştır. Buna karşılık A3 haplotipinin tromboz için 2.04 kat risk getirdiği ve A3 (4600A>G) alleli taşıyanların taşımayanlara göre daha yüksek sEPCR seviyesine sahip olduğu saptanmıştır. A3 haplotipi Türk populasyonunda hem trombotik riskle hem de artan sEPCR seviyesiyle ilişkilendirilebilir.

## INTRODUCTION

The protein C (PC) anticoagulant pathway is physiologicallyimportant to the regulation of coagulation.While the PC pathway plays a crucial role in controllingthrombosis, it also has anti-inflammatory functions [1-3].The essential components of this pathway include thrombin,thrombomodulin (TM), endothelial cell PC receptor(EPCR), PC, protein S, and protease-activated receptor 1(PAR-1).

The PC pathway is a natural anticoagulation mechanismthat prevents excessive thrombin generation. WhenThrombin binds to TM, PC is activated about 1000 timesfaster than free thrombin [1]. The thrombin-TM complexactivates PC. Activated PC (APC) requires protein S as acofactor. APC limits amplification and progression of thecoagulation cascade via proteolytic inactivation of factorVa (FVa) and factor FVIIIa [2]. PC activation increasesapproximately 20-fold in vivo when it is bound to EPCR[3].

EPCR binds to PC and also APC with high affinityand promotes PC activation on endothelium by increasincreasingthe catalytic efficiency of the thrombin-TM complex[4-6]. Although EPCR is an endothelial cell-specific typeI transmembrane protein, a soluble form of this receptorcirculates in plasma [7-9]. EPCR is similar to molecules ofthe class I major histocompatibility complex, in particularthe CD1-subfamily [10]. In addition to the extracellulardomains, EPCR has a transmembrane domain and a veryshort cytoplasmic tail.

Primary defects of the PC pathway increase the riskof venous thrombosis [10]. A metalloprotease cleaves theentire extracellular domain of EPCR from the cell membrane[11]. This cleavage results in the formation of solubleEPCR (sEPCR). sEPCR can bind both to PC and APCwith an affinity similar to that of intact membrane-boundEPCR. sEPCR inhibits PC activation by competing withthe membrane form of EPCR on vessel walls [9]. sEPCRalso inhibits inactivation of factor Va and APC anticoagulantactivity by blocking the interaction of APC and negativelycharged membrane surfaces [12].

Plasma sEPCR is elevated in patients with systemicinflammatory diseases [13]. sEPCR binding to APC blocks phospholipid interaction and alters the active site of APC.Inherited defects of the PC pathway are associated withan increased tendency for venous thromboembolism(VTE) [14], whereas low plasma sEPCR (sEPCR<30 ng/ml) appears to reduce the risk of thrombosis [15]. Highplasma sEPCR (sEPCR>130 ng/ml) leads to dysfunction ofEPCR-mediated coagulation [16].

The human EPCR gene is located on chromosome20q11.2 and is composed of 4 exons and 3 introns. Exon1 encodes the 5’ untranslated region (UTR) and the signalpeptide, exons 2 and 3 encode most of the extracellularregion of EPCR, and exon 4 encodes an additional10 residues of the extracellular region of EPCR, the transmembranedomain, the cytoplasmic tail, and the 3’ UTR[15,17]. In adults, EPCR is primarily located on the endothelialcells of large blood vessels, and exists in very lownumbers or is absent from the microvascular endotheliumof most tissues [18].

To date, more than 92 polymorphisms and 5 diseaserelatedmutations have been observed on the human EPCR gene [5,19,20]. Among these polymorphisms anddisease-related mutations a 23-bp duplication locatedbetween intron 2 and exon 3 (g.4189_4213dup TATCCACAGTTCCTCTGACCATCaccording to NT_011362.10)was reported to be associated with the risk of arterial andvenous thrombosis [21, 22]. A non-synonymous singlenucleotide polymorphism (SNP) in exon 4, rs867186(p.ser219gly; A4600G) (referred to as haplotype 3), isassociated with elevated plasma sEPCR and causes thrombosis[23]. On the other hand, another polymorphism inthe 3’ UTR of the gene, rs9574 (C4678G) (referred to ashaplotype A1), does not contribute to the risk of thrombosis[19,23,24,25]. This allele was also reported to be associatedwith a reduced risk of venous thrombosis [26]. Assuch, the present study aimed to investigate the effect ofEPCR gene polymorphisms on the plasma sEPCR level inpatients with venous thrombosis and healthy controls.

## MATERIALS AND METHODS

The study included 111 patients and 73 controls.The patient group included 64 females (57.6%) and 47males (42.4%), and the control group included 48 females(65.7%) and 25 males (n34.3%). Mean age in the controlgroup was 28.5 years, versus 26.01 years in the patientgroup. None of the control group participants had a personaland a family history of venous thrombosis. All participantspresented to Ankara University, School of Medicine,Department of Pediatric Molecular Genetics. All theparticipants provided written informed consent and thestudy was approved by the Ankara University School ofMedicine Ethics Committee.

Plasma sEPCR was measured via enzyme-linked immunosorbentassay (ELISA) (Diagnostica Stago Asserachrom,Asnieres, France) [27]. DNA was isolated via proteinase Kand phenol/chloroform extraction. All exons of the EPCR siggenewere screened using polymerase chain reaction (PCR)and the following primers that were designed in our laboratory;5’gccccctagtaggaaatga3’ and 5’gagatgtgcccccgactc3’for exon 1 (293 bp), 5’caggcctccaaagacttcat3’ and 5’cctactcacaggccaaggtc3’for exon 2 (264 bp), 5’gcaccctctctgcacagtc3’and 5’ccatccatttgtctggaacc3’ for exon 3 (384 bp), 5’taaacgggtccctttcctct3’ and 5’ctcccctccctcaaatcttc3’ for the1st region of exon 4 (384 bp), 5’caccagaaggtttggagtgac3’and 5’acgcctcaggtgattctgtc3’ for the 2nd region of exon 4(247 bp), and 5’ccatcctccaaagacagacag’ and 5’ccagaaattttgcaaagtgga3’for the 3rd region of exon 4 (278 bp).

Single strand conformation polymorphism (SSCP) wasperformed to determine if there was band pattern variationamong the participants. SSCP was performed in 143 of theparticipants to detect the polymorphisms. Sequencing ofthe EPCR gene was performed when SSCP yielded a differentband pattern (Beckman Coulter CEQ 8000, BeckmanCoulter, USA). Direct sequencing of exon 4 was performedto detect A4600G mutation, whereas sequencing and REanalysis were used to detect C4678G mutation. All theparticipants were genotyped using 4678G to C substitution,which creates a Ddel restriction site (5’C/TNAG 3’).The samples were incubated using Ddel restriction enzyme(Fermentas UAB, Vilnius, Lithuania) at 37 °C for 16 h, andthen the digestion products were electrophoresed on 3%agarose gel (Sigma, USA).Statistical analysis was performed using TADPOLEv.2.01 for Windows. The level of statistical significancewas set at P <0.05. The odds ratio (OR) and 95% confidenceinterval (CI) were calculated based on the logisticmodel. Allele frequencies were calculated via gene counting.In all these analyses the group with homozygous normal(4678 CC) served as the reference category to whichrisk was expressed.

## RESULTS

**Figure 1:**
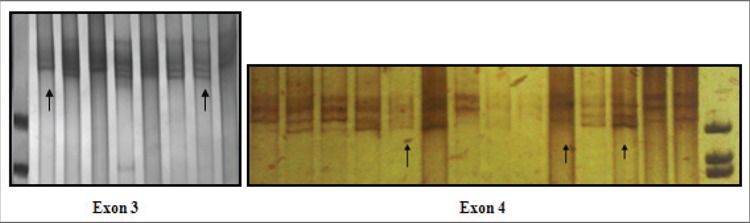
SSCPshows band patternvariation in exon 3and the first region ofexon 4.

**Figure 2:**
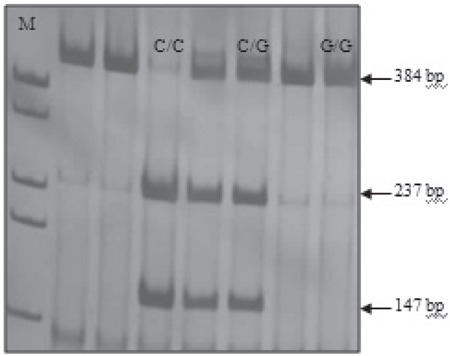
Restriction endonuclease analysis ofC4678G polymorphism.

**Figure 3:**
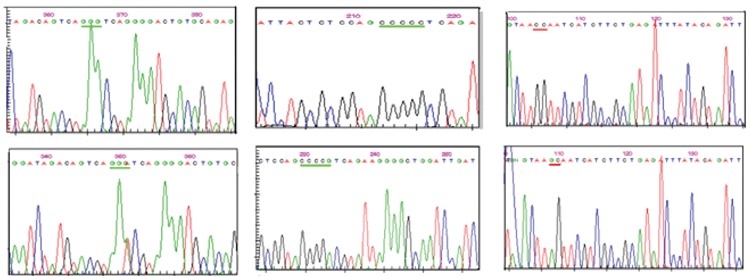
Sequencing results for C3998T(A), C4678G(B), and A4600G(C).

SSCP band pattern variation was noted in exons 3 andthe first region of exon 4(Figure 1). Three SNPs in exons3 and 4 that have been previously described; (C3998T)rs2069952 [31,32,33], located in intron 2; (C4678G;A1 haplotype)rs9574 [31,32,33], located in 3’UTR and (A4600G;p.ser219gly A3 haplotype) rs867186 [31,32,33] located inexon 4; were observed in our control and patient groups.Restriction endonuclease analysis of C4678G polymorphismis shown in Figure 2, and sequencing results forC3998T, C4678G, and A4600G are shown in Figure 3.SSCP, on the other hand, showed that there wasn’t anyband pattern variation in exon 2, or the 2nd and 3rd regionsof exon 4. In the present study plasma sEPCR levels of38-132 ng μL–1 were considered normal. sEPCR levelswere significantly higher in participants (patients andcontrols) carrying the A3 allele. Among the participantsthat carried the A1 allele, plasma sEPCR did not differ sigsernificantly(Table 1). There wasn’t a significant differencebetween controls and patients in the genotype frequencyof C3998T substitution (P = 0.58, OR = 0.5, CI = 0.22-1.38), indicating that the T allele was not associated withthe risk of venous thrombosis (P = 0.0009, OR = 0.43, CI =0.26-0.70). C4678G substitution was not a risk for venousthrombosis (P = 0.018, OR = 0.82, CI = 0.31-2.20); itmight actually have offered protection against thrombosis.The 4600G allele was associated with a 2.04-fold increasein the risk of venous thrombosis for patient group, as comparedto the control group, although the difference wasn’tstatistically significant (P = 0.81, OR = 2.04, CI = 0.20-20.15) (Tables 2,3,4). The A3 haplotype was associated withboth elevated plasma sEPCR and increased risk of venousthrombosis.

## DISCUSSION

Numerous variations in the EPCR gene have been reported. Any defect in the EPCR gene that leads to are duction in receptor expression or impaired receptor function may stimulate the development of thrombosis.A 23-bp duplication of nucleotides between 4189 and4213 at the genomic level, according to NT_011362.10(a number of previous publications has referred this as position 4031) [28], results in an early stop codon and synthesis of a truncated protein that is not expressed on endothelial surfaces [21]. This duplication is associated with an increase in the risk of arterial and venous thrombosis[21,22].

A polymorphism in exon 4 (A3 haplotype) encodes serine instead of glycine at codon position 219 (NM 006404.3);this occurs in the trans membrane region of EPCR [24].This haplotype is reported to be responsible for 86.5% ofthe variation in plasma sEPCR levels [15]. Several studies have examined the correlation between p.S219G polymorphism and the risk of thrombosis. Saposnik et al. reported that the incidence of p.S219G polymorphism in patients with venous thrombosis is higher than that in healt hycontrols [23]. Ireland et al. observed that p.S219G homozygosity was associated with a 3-fold increase in the risk of coronary heart disease [29]; however, 2 other studies did not observe a similar correlation between p.S219Gpolymorphism and increased risk of venous thrombosis[15,25]. In the present study another polymorphismwas identified—rs9574 (haplotype A1, C4678G)—that has been reported to be associated with high levels of circulating APC and a reduction in the risk of deep venousthrombosis (DVT) [15,26]. Saposnik et al. reported that the C4678G polymorphism had no effect on the plasmas EPCR level [23]. Furthermore, Saposnik et al. studied EPCR mRN A forms in eukaryote cells and compared the irexpression patterns based on cell genotype. They reported that there was a statistically significant higher quantity of mRN A that encoded a protein lacking the transmembrane domain in cells carrying A3 than in non-A3 carrying cells.They also reported that this EPCR protein was indeed synthesized and secreted, and that sEPCR was generated viaADAM17 cleavage [24].

Navarro et al. reported that mean age at initial onset of thrombosis was higher in non-carriers of the rs867186(A4600G) allele (44 ± 14 years) than in carriers (35 ± 8years), and that the probability of not having thrombosis at age 40 years was lower in prothromb in 20210A carriers with the EPCR A4600G allele. They also reported that the presence of the A4600G allele, plasma sEPCR >147 ngmL–1, and prothrombin >129% all were associated withan increase in the risk of thrombosis. The risk of venousthromboembolism is influenced by the prothrombin level and the EPCR A3 haplotype, due to their effect on sEPCR levels [30]. Smoking, body mass index, ABO blood groups,levels of FII, FV, FVII, FIX, FX, FXI, FXII, and FXIII A andB subunits, fibrinogen, protein S, and antithrombin do not have any effect on the plasma sEPCR level [15].

The results of the present study confirm that there isa strong correlation between the A3 haplotype and elevatedplasma sEPCR, as previously reported [15,23,29].This allele also increased the risk of venous thrombosis2-fold; however, there was a statistically significant difference between the patient and control groups, which might have been due to the small number of participants. The role of 3’UTR in RN A stability is known; as such,EPCR 3’UTR C4678G polymorphism (rs9574) mighthave an important thrombotic effect , but this hypothesismust be proven by large-scale studies. Moreover,C3998T polymorphism did not have a strong correlation with venous thrombosis in the present study. The present study’s findings are in agreement with those previous published concerning sEPCR and EPCR gene haplotypes;however, additional research is needed in order to clarify the effects of EPCR gene mutations on venous thrombosis and inflammation.

## CONFLICT OF INTEREST STATEMENT

The authors of this paper have no conflicts of interest,including specific financial interests, relationships, and/or affiliations relevant to the subject matter or materialsincluded.

## ACKNOWLEDGMENT

This study was supported by the Turkish Society of Hematology.

## Figures and Tables

**Table 1:**
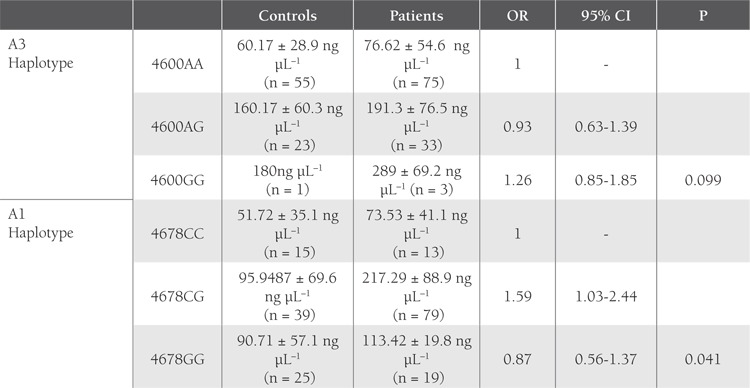
Mean Plasma sEPCR Level in the Participants with the A3 Allele (SNP no: rs867186) and A1 Allele (SNP no: rs9574) [31,32,33]

**Table 2:**
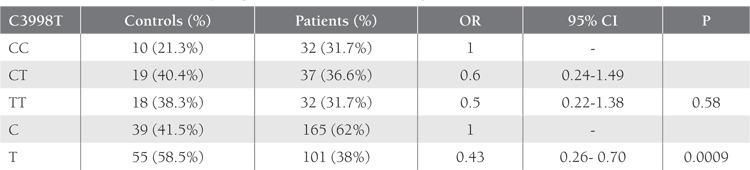
Distribution of C3998T Polymorphism at Intron 2 of the EPCR gene

**Table 3:**
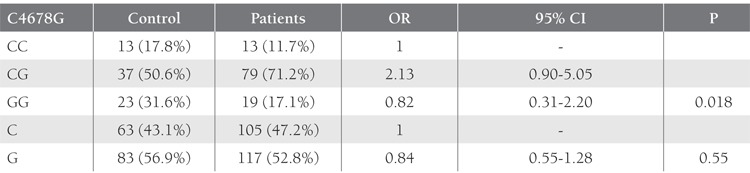
Distribution of C4678G Polymorphism (A1 Haplotype) at 3’UTR of the EPCR gene

**Table 4:**
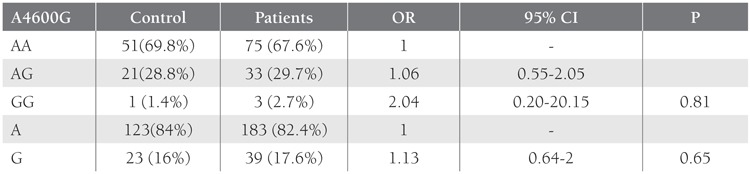
Distribution of A4600G Polymorphism (A3 Haplotype) at Exon 4 of the EPCR gene
